# A Treatise on the Principles and Practice of Medicine

**Published:** 1894-10

**Authors:** 


### Book Reviews.

A Treatise on the Principles and Practice of Medicine. Designed for the Use of Practitioners and Students of Medicine. By Austin Flint, M.D., LL.D., late Professor of Principles and Practice of Medicine in the Bellevue Hospital Medical College, New York City, etc. Seventh Edition, Thoroughly Revised by Frederick P. Henry, A.M., M.D., Professor of Principles and Practice of Medicine in Woman's Medical College of Pennsylvania, etc. Philadelphia. Lea Brothers & Co. 1894.

This book is before us. We look upon it with special pleasure, having been personally acquainted with the distinguished author of the original work, and having had the pleasure and profit (we hope) of his wonderful teaching. We have read in the New York *Medical Journal* the review of the book by his son. As to the omission of the section on general pathology, that may be well, and yet there is much in that for interest and instruction to the general practitioner. "All times are good when old," but the old times are sometimes better than the new. Of course there is much that has been learned by the medical profession in the study of therapeutics, and chemistry, and the use of the microscope, but we must not go too fast. The object is to cure the patient, and sometimes "our grandmothers knew better than we know." It is to be especially regretted, we think, that the "article on dyspepsia has been expunged." The editor says: "The term dyspepsia is a most serviceable cloak for ignorance, but has been worn threadbare and is about to be discarded." What is dyspepsia? Who knows? It is generally conceded to be an impairment of digestion, originating either in the stomach (gastric) or intestine, and due either to acute or chronic catarrh of these organs, to lack of tone without obvious inflammation (atonic), to reflex causes (ovarian disease, etc.), absence of the proper amount of acid (hydrochloric) in the stomach, etc. One must study the case, but the patient does not usually want the introduction of a tube into the stomach to "get some of the fluids to examine," etc.

Let any one read the article of Dr. Flint on this subject (dyspepsia), and he will find much in it to comfort him and his patient,—a thorough knowledge of the physiology of digestion, and therefore of dyspepsia—and many suggestions for the relief of the patient.

With these remarks we leave the book to the profession. It is a good book. We only regret what has been stated above.

